# Roles of tumor-associated macrophages in tumor progression: implications on therapeutic strategies

**DOI:** 10.1186/s40164-021-00252-z

**Published:** 2021-12-29

**Authors:** Shuangli Zhu, Ming Yi, Yuze Wu, Bing Dong, Kongming Wu

**Affiliations:** 1 Department of Oncology, Tongji Hospital of Tongji Medical College, Huazhong University of Science and Technology, Wuhan, 430030 China; 2 Department of Molecular Pathology, The Affiliated Cancer Hospital of Zhengzhou University & Henan Cancer Hospital, Zhengzhou, 450008 China

**Keywords:** Tumor-associated macrophages, Tumor progression, Therapeutic strategies

## Abstract

Macrophages are heterogeneous cells that present as different functional phenotypes due to their plasticity. They can be classified into two categories, namely M1- and M2-like macrophages, which are involved in processes as diverse as anti-tumor activity and immunosuppressive tumor promotion. Tumor-associated macrophages (TAMs) are defined as being of an M2-type and are considered as the active component in tumor microenvironment. TAMs are involved in multiple processes of tumor progression through the expression of cytokines, chemokines, growth factors, protein hydrolases and more, which lead to enhance tumor cell proliferation, angiogenesis, and immunosuppression, which in turn supports invasion and metastasis. It is assumed that the abundance of TAMs in major solid tumors is correlated to a negative patient prognosis. Because of the currently available data of the TAMs’ role in tumor development, these cells have emerged as a promising target for novel cancer treatment strategies. In this paper, we will briefly describe the origins and types of TAMs and will try to comprehensively show how TAMs contribute to tumorigenesis and disease progression. Finally, we will present the main TAM-based therapeutic strategies currently available.

## Introduction

Macrophages are present in various tissues and are involved in both innate and adaptive immunity. It is known that macrophages are phenotypically heterogeneous and functionally diverse [1]. At least two different populations can be distinguished: M1-(pro-inflammatory) and M2-like macrophages (anti-inflammatory and immunoregulatory) [2]. Their functional diversity is due to differences in phagocytosis, antigen presentation, and release of cytokines and complement components [3, 4].

Tumor-infiltrating macrophages (TAMs) are important cellular components of the tumor microenvironment (TME) [5]. The term “TAMs” mainly refers to macrophages differentiated into an M2-like phenotype because their functional characteristics more closely resemble an M2- rather than an M1-like phenotype [6, 7]. Tumor-derived cytokines and non-coding RNAs attribute to this transformation [8, 9]. Interestingly, Lu et al. found that OCT-4 not only induced stemness of lung cancer cells but also promoted M2 macrophage polarization through M-CSF secretion [10]. Pancreatic ductal adenocarcinoma (PDAC) cells with endothelial-mesenchymal transition (EMT) secreted HSP90 to induce macrophage M2-polarization and increase tumor growth through feedback regulation [11]. The recruitment of macrophages and activation in the TME is regulated via signals generated by tumor cells as well as host cells. Among the signals are C–C chemokine ligand 2 (CCL2), CCL5, colony-stimulating factor-1 (CSF-1), and granulocyte–macrophage colony-stimulating factor (GM-CSF) [12–15]. Communication within the TME is bidirectional because TAM signaling is involved in tumor initiation, accelerated disease progression and metastasis [16]. There is increasing evidence that TAMs influence various aspects of tumor progression such as tumor initiation, tumor angiogenesis, immunosuppression, invasion, and metastasis through the release of cytokines or growth factors into the TME [15, 17, 18].

A series of studies have shown that the abundance of TAMs was associated with negative prognosis in patients with solid tumors, except non-small cell lung cancer (NSCLC) [19]. Therefore, TAMs are considered a new potential target in cancer treatment. Elucidating the role of TAMs can provide useful insights for studying tumor pathogenesis and developing new therapeutic strategies. Currently, the main therapeutic strategies of targeting TAMs include targeting tumor angiogenesis, promoting TAM phagocytosis, reprogramming TAMs, blocking immune checkpoint inhibitors, and inhibiting TAM recruitment or direct depletion of TAMs [20, 21]. The dynamic changes, phenotypes, signaling pathways and functional states of TAMs in the process of tumorigenesis and development make TAMs a hot topic of research. In this paper, we briefly describe the origins and types of TAMs and will try to comprehensively show how TAMs contribute to tumorigenesis and disease progression. Finally, we will present the currently main TAM-based therapeutic strategies.

## TAM origins and types

### Origin

**Fig. 1:**
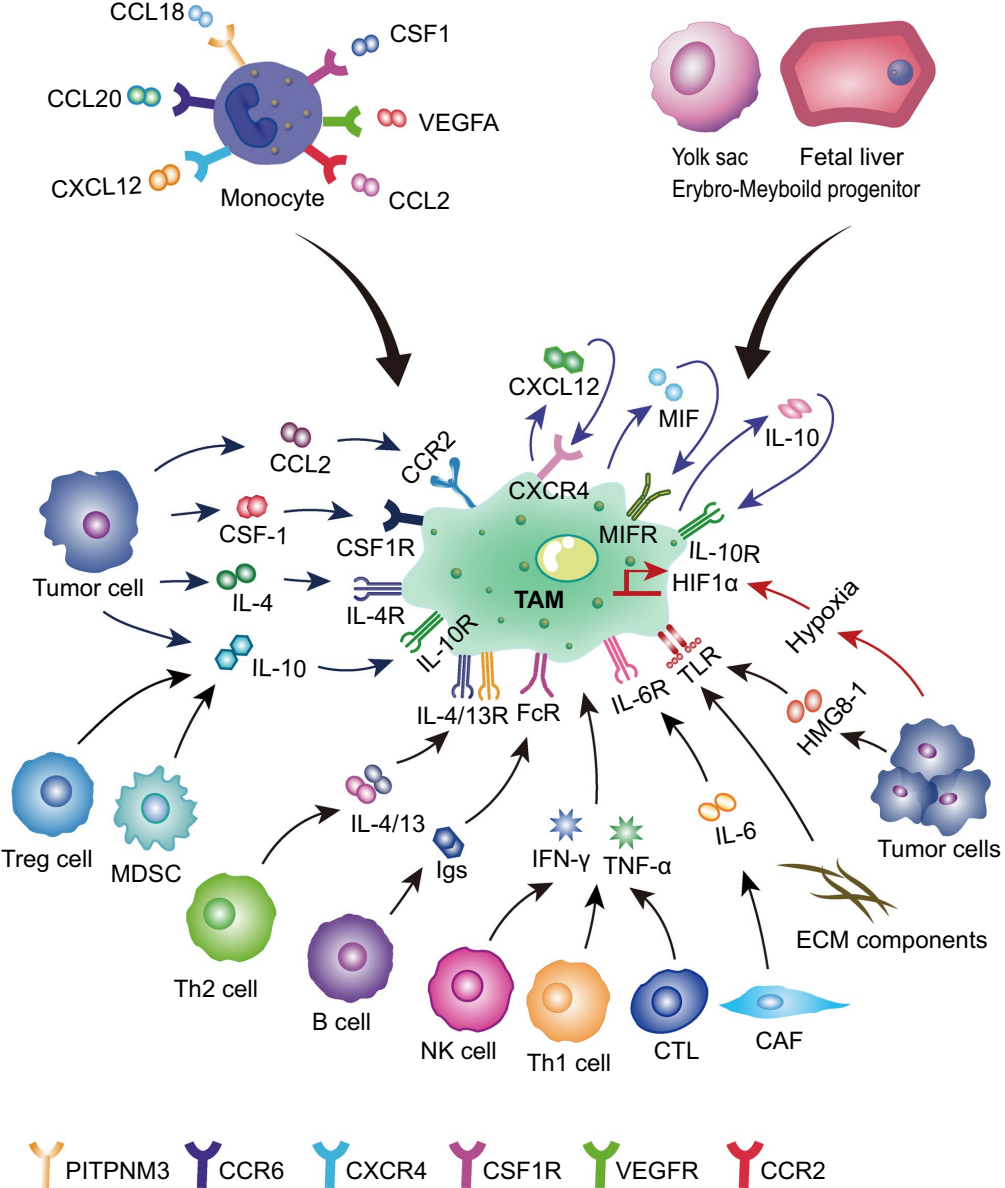
Origin of TAMs and their interaction with the TME. TAMs are mainly derived from bone marrow monocytes or erythroid myeloid progenitor cells in the yolk sac or fetal liver. Bone marrow monocytes are recruited and differentiated into TAMs by chemokines or cytokines released from tumor cells or stromal cells in the tumor microenvironment, such as CCL2, CSF-1, VEGFA, etc. TAMs are stimulated by CCL2, IL-4, and IL-10, secreted by tumor cells, and Igs, IL-10, and IL-4/13, secreted by immune cells (B cells, Treg cells, Th cells). Moreover, they can be activated by hypoxia, tumor-derived HMGB-1, and factors released by TAMs themselves (IL-10, MIF, and CXCL12). *TAMs* tumor-associated macrophages, *CCL2* C–C chemokine ligand 2, *CSF-1* colony-stimulating factor-1, *VEGFA* vascular endothelial growth factor A, *IL-4* interleukin-4, *IL-10* interleukin-10, *Treg cells* regulatory T cells, *Th cells* helper T cells, *MIF* macrophage migration inhibitory factor, *CXCL12* C-X-C motif chemokine ligand 12

Macrophages were first discovered in the late nineteenth century by Metchnikoff and were described as immune cells derived from the mononuclear phagocytic lineage present in most animals [22]. Due to their existence in many tissues, macrophages are commonly referred to according to their tissue contexts, such as microglia, Kupffer cells, and epidermal Langerhans cells. A more general term indicating their origin has also been coined: tissue-resident macrophages [23]. Macrophages are derived from both erythroid-myeloid progenitors (EMP) in the yolk sac and fetal liver and monocyte-producing macrophage/dendritic progenitors (MDP) of the bone marrow [24]. Macrophages have been shown to be present in many solid tumors and have therefore been termed tumor-associated macrophages [5]. Franklin et al. proposed two developmental pathways that may explain the occurrence of TAMs in tumor tissues: (1) tissue-resident macrophages of embryonic or monocyte origin may change their phenotype/activation status during tumorigenesis and have therefore been termed tissue-resident TAMs; (2) monocytes undergo a distinct stage of differentiation during tumor growth and eventually become macrophages which is why these cells have been termed tumor-induced TAMs. These two cell populations may coexist in a particular tumor of which tissue-resident TAMs may predominate in the early stages of tumor growth while tumor-induced TAMs are predominant in the later stages of the tumor. In addition, monocytes entering the tumor tissue may phenotypically change in response to TME without differentiating into TAMs which is why these cells are called tumor-induced effector monocytes [25]. In the mouse model, TAMs were mainly derived from bone marrow monocytes. These monocytes were recruited via inflammatory signals (e.g. CCL2, CCL18, CCL20, C-X-C motif chemokine ligand 12 (CXCL12), CSF1, and vascular endothelial growth factor A (VEGFA)) released by cancer cells to primary or metastasis tumors where they differentiated into TAMs, further facilitating disease progression and metastasis [26, 27] (Fig. 1).

### Types

According to the activation type and the different roles in TME, macrophages are usually divided into two types, M1 with a classical activation and M2 with an alternate activation pathway [1, 28]. Once M1-phenotype macrophages have activated themselves through cytokines such as interferon (IFN)-γ, tumor necrosis factor (TNF)-α, or lipopolysaccharide (LPS) [29, 30], they further produce pro-inflammatory and immune-stimulating cytokines and participate in the anti-infection response together with helper T cells 1 (Th1). In addition, M1-type cells can kill target cells by phagocytosis [31–33]. Finally, M1 cells also express nitric oxide synthase (iNOS), reactive oxygen species (ROS) [34–36], and cytokines such as interleukin-12 (IL-12) [37]. M2-type cells are mainly activated by Th2-related cytokines (e.g. IL-4, IL-10, and IL-13) and suppress T cell responses as well as promote tumor cell growth, invasion, and metastasis [1, 31–33]. In addition, they express scavenger receptors or cell differentiation (CD) markers (CD68, CD163, CD206) [38] that are associated with a high expression of IL-10, IL-1β, VEGF, and matrix metalloproteinases (MMP) [39, 40]. It is worth noting that M2 cells can be divided into more subtypes (M2a, M2b, M2c, M2d) [38, 41]. Recent research has shown that TAMs correspond to a state located between M1 and M2 [42], however, based on the role in TME, they more closely resemble an M2-phenotype [1, 43].

## The role of TAMs in tumor progression

**Fig. 2:**
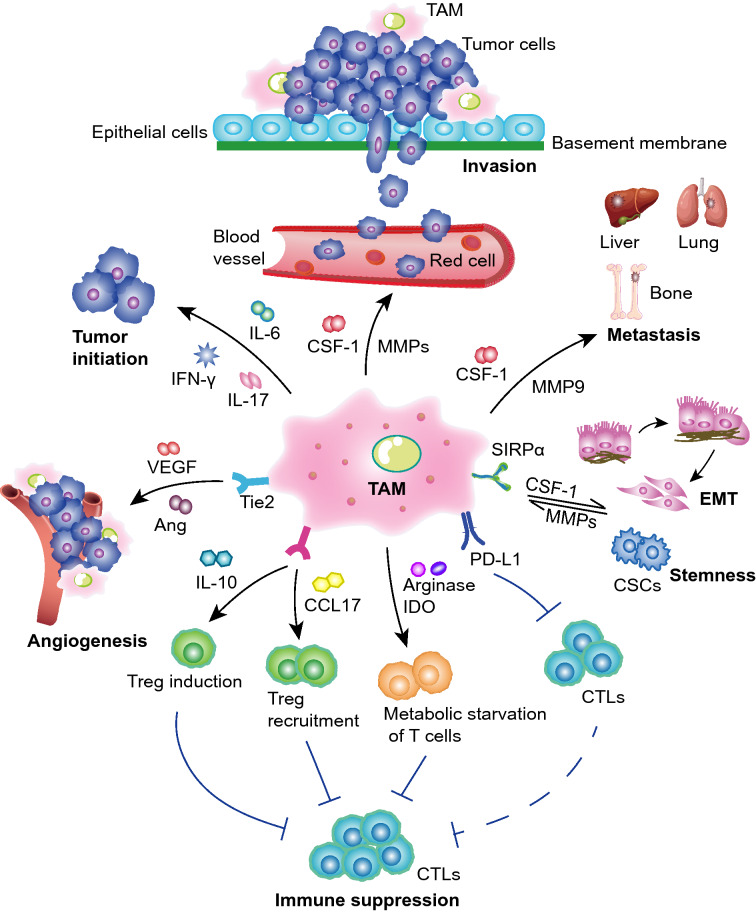
The effects of TAMs on tumor progression. The schematic diagram shows that TAMs promote tumorigenesis, angiogenesis, invasion, metastasis, epithelial-mesenchymal transformation (EMT) and the acquisition of stem cell characteristics. TAMs suppress the immune response through secretion of certain factors or proteases. *TAMs* tumor-associated macrophages, *IL-6* interleukin-6, *IL-17* interleukin-17, *IFN-γ* Interferon-γ, *VEGF* vascular endothelial growth factor, *Ang* angiotensin, *IL-10* interleukin-10, *CCL17* C–C chemokine ligand 17, *IDO 1/2* indoleamine 2,3-dioxygenase 1/2, *PD-L1*, programmed cell death ligand 1, *CTLs* CD8^+^ cytotoxic T lymphocytes, *CSCs* cancer stem cells, *MMPs* metalloproteinases, *CSF-1* colony-stimulating factor-1, *EMT* epithelial mesenchymal transformation, *SIRP α* signal-regulatory protein α, *MMP2/3/7/9* metalloproteinase 2/3/7/9

Immune cells are among the main components of TME and include macrophages, T cells, natural killer cells (NK cells), dendritic cells, and more. TAMs, as the major immunosuppressive cells, have a wide range of effects on TME through the synthesis and secretion of various cellular factors [44] (Fig. 2).

### Promotion of cancer initiation

Researchers found abundant inflammatory cells in tumor biopsy samples which renders it likely that chronic inflammation may be associated with tumor initiation [45, 46]. Expectedly, this has been shown in cases of colon and gastric cancer [47]. This can be explained by findings showing that chronic inflammation (persistent infection, repeated exposure to irritants, autoimmune diseases) or oncogene activation can lead to the expression of pro-inflammatory transcription factors such as nuclear factor-κB (NF-κB), signal transducer and activator of transcription 3 (STAT3), and hypoxia inducible factor 1 α (HIF-1α). After these factors have been activated, they could lead to the recruitment of macrophages mediated by the expression of cytokines and chemokines (TNF-α and IL-6) of cancer cells [48]. Macrophages can produce proinflammatory mediators such as IL-6, TNF, IFN-γ, growth factors, including epidermal growth factor (EGF) and Wnt, proteases, ROS, and nitrogen substances that may produce a mutagenic microenvironment, further facilitating cancer initiation [20, 49, 50]. Grivennikov et al. proved that TAM-derived IL-17 and IL-23 were connected to the growth and progression of colon cancer [51]. The study by Kong et al. suggested that TAM-derived IL-6 could facilitate the initiation and development of hepatocellular carcinoma (HCC) by activating the STAT3 signaling pathway [52]. In summary, TAMs may have a plethora of effects in the course of the development and occurrence of cancer.

### Promotion of angiogenesis

Besides their capability of promoting cancer-related inflammation processes, TAMs can also directly affect tumor growth via the promotion of angiogenesis. Angiogenesis is necessary to meet the cancer cells’ growth demands reflected by an increased need for oxygen and nutrients [53]. The neovascularization is also crucial for tumor invasion and metastasis, referring to many factors, such as hypoxia, hyperosmotic pressure and angiogenic factors such as VEGF, transforming growth factor β (TGF-β), cyclooxygenase-2 (COX-2), placenta growth factor (PGF), fibroblast growth factor (FGF) [54], epidermal growth factor (EGF), platelet-derived growth factor (PDGF), angiotensin (Ang), and chemokines [55–57]. HIF expression was associated with the stimulation of neovascularization and caused tumor cells to produce pro-angiogenic factors (e.g. VEGF-A, FGF-2) in hypoxic areas. In line with these findings is the fact that HIF-1ɑ could upregulate the expression of VEGF in highly hypoxic glioma [58]. Yin et al. reported that EGF secreted by TAMs could activate the tumor cells' surface EGFR and further upregulate VEGF/VEGFR signaling which supported ovarian cancer cell proliferation and invasion [56]. Cui et al. showed that glioblastoma-induced TAMs could promote tumor angiogenesis through elevating the secretion of TGF-β1 and IL-10 resulting in endothelial cell proliferation [59]. TAMs express proteases, such as metalloproteinase-9 (MMP-9), MMP-2, and MMP-3, and are able to degrade extracellular matrix (ECM), thereby further indirectly facilitating angiogenic invasion [60]. The Wnt/β-catenin signaling pathway is involved in proliferation, apoptosis, invasion, and metastasis [61] and it has been proved that an aberrant Wnt/β-catenin signaling cascade facilitated cancer development [62]. Wnt7b (Wnt family ligand) expression was upregulated by TAMs which could promote tumor neovascularization [63]. Yeo et al. could show that Wnt7b could stimulate VEGF production that in turn triggered angiogenesis switches and further targeted endothelial cells to contribute to tumor growth, tumor invasion, and metastasis [64]. It has been demonstrated that an upregulated expression of VEGFA in TIE2(hi)-derived macrophages induced the proliferation of endothelial cells and by this, led to tumor neovascularization [65]. The above studies show that the TAMs-driven tumor angiogenesis and the associated promotion in tumor progression provide a theoretical basis for an anti-angiogenesis therapy strategy by targeting the TAMs.

### Promotion of invasion and metastasis

Cancer cell invasion and metastasis are the major causes of death. Cancer cells acquire the ability to move and release degradative enzymes which permit tumor cells to break away from the primary tumor, they then colonize distant sites where they establish new tumors [66]. EMT is a process during which epithelial cells acquire mesenchymal character and through which malignant biological features are conferred, including invasion and metastasis [67, 68]. Recently, a series of studies demonstrated that TAMs take part in the regulation of the EMT process and facilitate metastasis [69–72]. The work by Wei et al. indicated that TAMs interfere with JAK2/STAT3/miR-506-3p/FoxQ1 regulation which increases colorectal cancer (CRC) cell invasion and metastasis capability via EMT induction. Moreover, the activation of this axis could result in the generation of CCL2 and thereby facilitate the recruitment of macrophages [73]. Tu et al. also found that a high TCF4 expression was associated with recruitment and polarization of macrophages in the metastasis sites. Moreover, the CCL2/CCR2 signaling pathway was shown to enhance metastasis [74]. Lee et al. performed a coculture study of non-neoplastic MCF10A human breast epithelial cells with TAMs. The results showed that TAMs could secrete CCL2 that led to the upregulation of endoplasmic reticulum oxidoreductase (ERO)1-α as well as MMP-9, and further inducing MCF10A acquired EMT invasive phenotype [75]. Similarly, CCL5 released by TAMs could significantly promote invasion, metastasis, and EMT of prostate cancer cells by activating the β-catenin/STAT3 signaling pathway [76]. Malignant phyllodes tumor recruited and repolarized TAMs through CCL5 binding to CCR5 in macrophages and then activated the AKT signaling pathway. Moreover, TAM-generated CCL18 bound PIPTNM3 (the receptor of myofibroblast) that further facilitated differentiation and the invasion of myofibroblast [77]. Lan et al. found that CCL26 together with CCR3 could induce the infiltration of TAMs. Further analysis showed that phosphatase of regenerating liver-3 (PRL-3) upregulated CCL26 which led to TAM infiltration and promotion of invasion and metastasis in CRC [78]. The conditioned medium from TAMs co-cultured with NSCLC cells facilitated tumor cells invasion via EMT and the upregulation of αB-Crystallin (CRYAB), and further inducing lung cancer metastasis in vivo [79]. Han et al. suggested that TAMs facilitated osteosarcoma metastasis and invasion through upregulating the expression of COX-2, MMP9, and phosphorylated STAT3 leading to EMT induction [80]. TAMs can express a variety of factors to induce EMT, such as TGF-β and IL-6. It has also been shown that TAMs secreted EGF to activate the EGFR/ERK1/2 signal pathway in cancer cells which might promote EMT [81].

### Immune suppression

TAMs express programmed cell death 1 (PD-1) and CTLA-4 receptor ligands, such as PD-L1 and B7-H1 (CD80), which have been shown to inhibit the cytotoxicity of T cells and NK cells [82, 83]. TAM-born chemokines and cytokines can inhibit the antitumor effect of tumor-infiltrating T cells and NK cells, and cooperate with bone marrow-derived suppressor cells (MDSCs), tumor-related dendritic cells, and neutrophils to form an inhibitory TME [84, 85]. The TAM-produced chemokines IL-10 and TNF-α induce the expression of PD-L1 and further inhibit the antitumor T cell activity [86]. TAMs can also inhibit the proliferative ability of CD8^+^ T cells via the release of arginase1, iNOS, oxygen radicals, or nitrogen species [87–89]. TAMs secrete anti-inflammatory chemokines to recruit regulatory T (Treg) cells, such as CCL2, CCL3, CCL4, CCL5, and CCL20 [90]. Moreover, TAMs mediate Tregs via generating CCL22 to suppress T cell-specific activity and further promote the growth of cancer cells [91]. Microenvironment characterization by multi-omics signatures performed by Liu et al. proved that TAM enriched HCC tissues were associated with immunosuppression [92]. Eisinger et al. demonstrated that targeting an immune-suppressive TAM subtype by specific antibodies against the scavenger receptor MARCO resulted in the phenotypic conversion of TAMs into proinflammatory TAMs that recruited and activated more NK cells to enhance the TNF-related apoptosis-inducing ligand (TRAIL) mediated effect of tumor cell killing [93]. Petty et al. stressed that hedgehog (Hh) signaling induced the polarization of TAMs through inhibiting the TAM generation of CXCL9 and CXCL10 which suppressed the recruitment of CD8^+^ T cells [94]. In conclusion, these findings support the immunomodulatory role of TAMs that may promote tumor progression by modulating the immune response and promoting immune evasion.

### Sustaining the activity of cancer stem cells

A cancer stem cell (CSC) is defined as a cell in tumor tissue that can self-renew to generate a heterogeneous set of tumor cells [95, 96]. Wan et al. found that TAMs could secrete IL-6 by STAT3 signaling to facilitate the expansion of HCC stem cells [97]. TAMs produce chemokines, such as CXCL8 and CXCL12, that are able to program cancer cells in a way that they acquire a CSC-like character and that maintain stemness in oral squamous cell carcinoma, HCC, and renal cell carcinoma [98–100]. Gomez et al. proved that head and neck squamous cell carcinoma-derived TAMs upregulated the interaction of hyaluronic acid (HA) (the ligand of CD44)-CD44 by HAS2. Once HA was bound to CD44, it increased stemness through activating the PI3K-4EBP1-SOX2 signaling pathway [101, 102]. Milk-fat globule-epidermal growth factor-VIII (MFG-E8) was released by TAMs which activated the STAT3 and the Sonic Hedgehog signaling pathway in CSCs, whereupon the cells, endowed with these new capabilities, displayed drug resistance and increased tumorigenicity [103]. TAMs could significantly upregulate the S100 calcium-binding protein A9, an inflammation-related secreted protein linked to poor survival in HCC patients that reinforced stem cell-like properties by activating NF-kB signaling [104]. Moreover, in pancreatic cancer, it was shown that TAMs could support the stemness of cancer cells by inducing the TGF-β1/Smad2/3 [105], and activating ERK1/2 pathway in glioblastoma [106]. A significant amount of in vivo and in vitro experiments suggests that the inhibition of the WNT/β-catenin pathway could weaken the TAM-induced upregulation of CSC stemness in HCC [107] and lymphoma [108]. These results strongly support the notion that TAMs support the induction, maintenance, and expansion of CSC and other stem cell subtypes (such as mesenchymal stem cells) in TME.

## Chemotherapeutic and radiotherapeutic resistance of TAMs

Radiotherapy and chemotherapy are common cancer treatments and the roles of TAMs in these therapies have been studied extensively. It was reported that TAMs could reduce the efficacy of cancer chemotherapy. TAM-born CCL5, which induced the activation of STAT3 and further mediated the upregulation of transcription factor Nanog, finally resulted in chemotherapeutic drug resistance [109]. Guan et al. found that prostate cancer cells increased the secretion of CXCL12 by TAMs after combined therapy of docetaxel together with androgen deprivation, which further facilitated cancer cell survival and led to a decreased response towards chemotherapy via CXCR4 activation [110]. EGFR-TKI is a new method for the treatment of advanced NSCLC [111]. Chung et al. investigated 206 cases of NSCLC patients who received EGFR-TKI treatment and found that TAM counts were significantly higher in patients with progressive disease than in those with non-progressive disease. Moreover, high TAM counts were significantly associated with lower progression-free survival and overall survival, suggesting that TAMs are related to reduced treatment responsiveness after EGFR-TKI administration [112].

In contrast, TAMs could enhance the effect of radiotherapy. TAMs are increasingly recruited into tumors after radiotherapy and can modulate the response of tumor cells to treatment [113]. Stafford et al. clarified that the CSF-1R inhibitor PLX3397 could prevent myeloid monocyte differentiation into TAMs, and improve the response of glioblastoma towards ionizing radiation treatment which further delays the recurrence of glioblastoma [114]. Rahal et al. found that PM37 could block IL-4/IL-13-mediated STAT6 tyrosine phosphorylation and decreased TAM–mediated radioresistance of inflammatory breast cancer cells by down-regulation of TAM–induced protein kinase C zeta [115]. Moreover, other studies demonstrated that targeting TAM or TAM-related signaling pathways could improve the radiotherapy effect [116–119].

In summary, TAMs are a double-edged sword since sometimes they promote tumor clearance whereas sometimes they accelerate cancer progression and treatment resistance. Therefore, the elucidation of their functions in tumorigenesis requires further exploration.

## Treatment strategies against TAMs

Many studies have shown that TAMs are correlated with a negative tumor prognosis in most human tumors [42] such as breast cancer, gastric cancer, and Hodgkin lymphoma [120–123]. Therefore, targeting TAMs is a potential strategy for cancer treatment **(**Table 1). In the following paragraphs, we summarized some aspects of this strategy.

**Table 1:** Clinical trials on TAMs (from https://clinicaltrials.gov/)

Action	Agent	Combination	Target	Status	Tumor type	Clinical trial number
Inhibiting the recruitment of TAMs	Carlumab	NA	CCL2	Completed II	Prostate cancer	NCT00992186
	CNTO 888(mAb)	Gemcitabine Paclitaxel and carboplatin docetaxel	CCL2	Completed I	Solid tumors	NCT01204996
	CNTO 888(mAb)	NA	CCL2	Completed I	Solid tumors	NCT00537368
	PF-04136309	FOLFIRINOX*	CCR2	Completed I	Pancreatic neoplasms	NCT01413022
	MLN1202	NA	CCR2	Completed II	Bone metastases	NCT01015560
Activating TAMs	MCS110	Carboplatin and Gemcitabine	CSF-1	Completed II	Triple-negative breast cancer	NCT02435680
	MCS110	Placebo	CSF-1	Completed II	Cancer	NCT01643850
	MCS110	NA	CSF-1	Terminated I/II	Prostate cancer, Bone Metastases	NCT00757757
	IMC-CS4 (LY3022855)	NA	CSF-1R	Completed I	Solid tumors	NCT01346358
	IMC-CS4	NA	CSF-1R	Completed I	Breast or Prostate cancer	NCT02265536
	AMG 820	NA	CSF-1R	Completed I	Solid tumors	NCT01444404
	AMG 820	pembrolizumab	CSF-1R	Completed I/II	Pancreatic cancer, Colorectal cancer, Non-small cell lung cancer	NCT02713529
	Emactuzumab	Atezolizumab	CSF-1R PD-L1	Completed Ib	Solid tumors	NCT02323191
	ARRY-382	Pembrolizumab	CSF-1R PD-1	Completed I b/II	Solid tumors	NCT02880371
	Pexidartinib	Durvalumab	CSF-1R PD-L1	Completed I	Pancreatic or Colorectal cancers	NCT02777710
	SNDX-6352	Durvalumab	CSF-1R PD-L1	Active, not recruiting I	Solid tumors	NCT03238027
	BLZ945	PDR001	CSF-1R PD-1	Active, not recruiting I/II	Solid tumors	NCT02829723
	Cabiralizumab (BMS-986227, FPA008)	Nivolumab (BMS-936558)	CSF-1R PD-1	Completed I	Malignancies	NCT03158272
	Trabectedin	Durvalumab (MEDI4736)	PD-L1	Active, not recruiting Ib	Soft-tissue sarcomas and ovarian carcinomas	NCT03085225
	PLX7486	NA	CSF-1R	Terminated I	Solid tumors	NCT01804530
	PLX3397 (Pexidartinib)	NA	CSF-1R	Terminated II	Glioblastoma	NCT01349036
	PLX3397	NA	CSF-1R	Completed II	Hodgkin lymphoma	NCT01217229
	PLX3397	NA	CSF-1R	Terminated II	Prostate cancer	NCT01499043
	PLX3397	Sirolimus	CSF-1R	Recruiting I Recruiting II	Sarcoma Malignant Peripheral Nerve Sheath Tumors	NCT02584647
	PLX3397	Pembrolizumab	CSF-1R	Terminated I/II	Tumors*	NCT02452424
	PLX3397	Eribulin	CSF-1R	Completed I b/II	Breast cancer	NCT01596751
	PLX3397(Turalio)	NA		Recruiting I/II	Leukemias, Solid tumors	NCT02390752
	PLX3397 (Pexidartinib)	Paclitaxel (Onxol)		Completed I	Solid tumors	NCT01525602
	Alemtuzumab	NA	CD52	Terminated I	Ovarian/primary peritoneal cancer	NCT00637390
	Alemtuzumab	fludarabine and cyclophosphamide	CD52	Completed II	Kidney cancer	NCT00073879
Reprogramming TAMs	Chi Lob 7/4	NA	CD40	Completed I	Malignancies	NCT01561911
	GM.CD40L* Vaccine	CCL21	CD40	Completed I/II	Lung cancer, Adenocarcinoma	NCT01433172
	CP-870,893*	NA	CD40	Completed I	Advanced solid tumors	NCT02225002
	CP-870,893	Tremelimumab*	CD40	Completed I	Melanoma	NCT01103635
	CP-870,893	Paclitaxel + Carboplatin	CD40	Completed I	Neoplasms	NCT00607048
	CP-870,893	Gemcitabine	CD40	Completed I	Adenocarcinoma pancreas	NCT01456585
	RO7009789	Emactuzumab (RO5509554)	CD40	Completed I	Neoplasms	NCT02760797
	RO7009789	nab-paclitaxel, gemcitabine	CD40	Completed I	Pancreatic cancer	NCT02588443
	RO7009789	Vanucizumab, Bevacizumab	CD40	Completed I	Solid tumors	NCT02665416
	Selicrelumab (RO7009789)	Atezolizumab	CD40 PD-1	Completed I	Solid tumors	NCT02304393
	APX005M	Nivolumab	CD40	Completed I/II	Non-small cell lung cancer, Metastatic Melanoma	NCT03123783
	IPI-549	Nivolumab	PI3K-γ PD-1	Active, not recruiting I	Solid tumors, non-small cell lung cancer, melanoma, breast cancer	NCT02637531
	TTI-621	PD-1/PD-L1 Inhibitor* pegylated interferon-α2a talimogene laherparepvec (T-Vec) radiation	SIRPα-IgG1 Fc	Terminated I	Solid Tumors, Mycosis fungoides, Melanoma, Merkel-cell carcinoma, Squamous cell carcinoma, Breast carcinoma, Human papillomavirus-related malignant neoplasm, Soft tissue sarcoma	NCT02890368
	Hu5F9-G4	Atezolizumab	CD47	Completed I	Acute myeloid leukemia	NCT03922477
	CC-90002	NA	CD47	Terminated I	Leukemia, Myeloid, Acute myelodysplastic syndromes	NCT02641002
	CC-90002	Rituximab	CD47	Completed I	Hematologic neoplasms	NCT02367196
	GSK3145095	pembrolizumab	RIP	Terminated II	Neoplasms, Pancreatic	NCT03681951
	NKTR-262	Bempegaldesleukin (NKTR-214) Nivolumab	TLR7/8 PD-1	Active, not recruiting I/II	Solid tumors	NCT03435640
	WP1066	NA	STAT3	Active, not recruiting I	Glioma and Brain metastasis from melanoma	NCT01904123
	AZD9150 (ISIS 481464)	NA	STAT3	Completed I/Ib	Advanced/Metastatic Hepatocellular carcinoma	NCT01839604
	Imprime PGG	Cetuximab	MAPK	Completed II	Colorectal Cancer	NCT00912327
	Immunological Adjuvant OPT-821	β-glucan		Active, not recruiting I/II	Neuroblastoma	NCT00911560
	β-Glucan	Anti-GD2 Monoclonal Antibody 3F8		Active, not recruiting I	Neuroblastoma	NCT00492167

FOLFIRINOX*: fluorouracil, leucovorin calcium, irinotecan hydrochloride, and oxaliplatin

Tumors*: Melanoma, Non-small Cell Lung Cancer, Squamous Cell Carcinoma of the Head and Neck, Gastrointestinal Stromal Tumor (GIST), Ovarian Cancer

GM.CD40L*: GM-CSF-Producing and CD40L-Expressing Bystander Cell Line

Tremelimumab*: Blocking Anti-CTLA-4 Antibody

CP-870,893*: Agonist Anti-CD40 Antibody

PD-1/PD-L1 Inhibitor*: nivolumab, pembrolizumab, durvalumab, avelumab, or atezolizumab

### Targeting angiogenesis

It could be shown that dual inhibition of VEGF/Ang-2 could prolong the survival of preclinical glioblastoma models through decreasing tumor burden, improving the vascular morphological normalization, and reprogramming TAMs to an M1-phenotype [124]. The HIF-1 pathway is stimulated by hypoxia and treatment with HIF-1 inhibitors led to the influx of CD11b^+^ bone marrow monocytes as well as the development of a tumor vascular system, both of which ultimately inhibited tumor cell regeneration [125]. However, understanding its specific anti-angiogenesis mechanism is useful to develop additional therapeutic strategies.

### Targeting phagocytosis of TAMs

CD47, a transmembrane glycoprotein expressed on cancer cells, serves as a “don't eat me" signal. CD47 combined with the signal-regulatory protein (SIRP) α (CD172a or SHPS-1) on macrophages could inhibit TAM phagocytosis [126, 127]. A large number of studies have shown that monoclonal antibodies were able to disrupt the CD47-SIRP 1α signaling pathway. These antibodies were capable of enhancing the phagocytosis of TAMs, increasing the number of tumor-infiltrating immune cells, suppressing the progression of tumor cells and hematological malignancies [128–130]. When the blockade of this signaling pathway was combined with mAbs targeting different tumor antigens, such as cetuximab, trastuzumab, and rituximab, the effect of cancer immunotherapies was even more pronounced [131, 132]. Clinical trials were developed using anti-CD47 antibodies (Hu5F-G4 [133], CC-90002, SRF231, and IBI188) and SIRFα-Fc fusion proteins (TTI-621, ALX148, SHR-1603) [134]. Golubovskaya et al. designed a CD47^+^-CAR (chimeric antigen receptor)-T cell able of targeting various cancer cells. It could be demonstrated that the CAR T cells could effectively kill ovarian, pancreatic, and cervical cancer cell lines, and moreover were able to generate IL-2 that is positively associated with the expression of CD47 [135]. In addition, Ferlin et al. suggested that targeting CD47 with a bispecific antibody might reinforce the anti-tumor activity and limit the toxicity in vivo [136]. To reduce off-target toxicity, engineered high-affinity CD47 extracellular domain-targeted blocking SIRPα, such as engineered SIRPα without Fc fusion, is now being developed [137].

### Targeting TAM-associated immune checkpoints

Nowadays, checkpoint blockade inhibitors as a part of cancer immunotherapy are widely accepted by clinicians [138]. It is known that clinically relevant targets comprise PD-1/PD-L1 and CTLA-4. Anti-PD-1/PD-L1 mainly exert their effects by facilitating the activation of tumor-specific cytotoxic T cells [139–141].

The expression of PD-1 is negatively related to the capability of TAM phagocytosis and blocking PD-1/PD-L1 in vivo increased the phagocytosis of TAMs, decreased the growth of the tumor, and further prolonged the survival time of the mice by depending on macrophages [142]. Xiao et al. reported that TAMs in HCC tissues had a high expression of Siglec-10 with an associated predicted poor prognosis. Blocking Siglec-10high TAMs improved immunotherapy against HCC [143]. Xiong et al. proved that anti-PD-L1 treatment led to a transformation of TAMs to an M1-phenotype by increasing the IFN-γ levels [144]. Su et al. found that macrophages following antibody-dependent cellular phagocytosis (ADCP) could inhibit the antibody-dependent cellular cytotoxicity (ADCC) that was mediated by NK cells, and also suppressed T cell-mediated cytotoxicity in breast cancer and lymphoma. Moreover, they also found that after anti-HER2 antibody administration that was combined with PD-L1 and indoleamine 2,3-dioxygenase (IDO) inhibitors, reinforced the therapeutic efficacy of anti-tumor immunity and anti-HER2 efficacy in mouse models [145].

Viitala et al. proposed that the macrophage scavenger receptor common lymphatic endothelial and vascular endothelial receptor-1 (Clever-1, also named Stabilin-1 or Feel-1) expressed by TAMs can be defined as a checkpoint receptor because Clever-1 gene defects cause immune stimulation of TAMs and concomitant activation of endogenous anti-tumor CD8^+^ T cells. Therefore, an immunotherapeutic blockage of Clever-1 could achieve a similar effect as a PD-1 checkpoint inhibitor administration. Moreover, the combination of an anti-Clever-1/anti-PD-1 inhibitor has a synergistic effect on aggressive tumors [146]. Zhou et al. hypothesized that blocking the phagocytic receptor MerTK leads to an apoptotic cell accumulation in tumors and triggers an IFN-α response, leading to a further increase in the anti-tumor immune response. The treatment of tumor-bearing mice showed that administration of an anti-MerTK antibody resulted in T cell activation which creates synergies in combination with an anti-PD-1/ PD-L1 administration [147]. Clinical trials evaluating the treatment with checkpoint inhibitors and anti-TAM agents (for example, anti-CSF-1R antibodies) are ongoing [20].

### TAM reprogramming

M1-like macrophages display anti-tumor activity, therefore, reprogramming tumor-promoting M2-like macrophages into tumor-killing M1-like macrophages seems to be a potential strategy in cancer therapy. This strategy mainly involves the activation of CD206 and Toll-like receptors (TLRs). RP-182-mediated activation of CD206 (mannose receptor) in M2-like TAMs induced endocytosis, phagosome lysosome formation, and autophagy, which resulted in a transformation of M2-like TAMs into M1-like TAMs and an associated enhancement of cancer cell phagocytosis [148]. Wang et al. reported that upregulation of serine/threonine-protein kinase 1 in TAMs of PDA tissues contributed to immune tolerance. Administration of RIP1 inhibitor in a mouse model led to the activation of CTL and the differentiation of Th cells to a hybrid Th1/ Th17 phenotype. Further mechanistic analysis proved that RIP1 inhibitor reprogrammed TAMs to an MHCIIhiTNFα^+^IFNγ^+^ phenotype via STAT1 [149].

Toll-like receptors (TLRs), a receptor of transmembrane pattern recognition, play their roles in innate immunity via recognizing pathogen-associated molecular patterns (PAMPs) [150]. TLR agonist administration (TLR7 agonist imiquimod, TLR9 CpG-oligonucleotide) resulted in the polarization of pro-inflammatory M1-macrophages by activating NF-κB [151]. So far, the TLR7 agonist imiquimod is the FDA-approved TLR agonist for clinical application which has shown significant anti-tumor activity in preclinical models of melanoma [152, 153]. Oya et al. showed that imiquimod induced an anti-tumor response via upregulating interferon γ (IFN-γ) expression in CD8^+^ T cells, however, imiquimod also enhanced the PD-1 inhibitory signaling resulting in T cell exhaustion. Combined treatment of imiquimod together with anti-PD-1 antibodies revealed a more obvious anti-tumor effect than each monotherapy [154]. The TLR7/8 agonist 3M-052 enhanced anti-tumor immunity by reprogramming TAMs to an M1-phenotype and the combined therapy with 3 M-052 and anti-CTLA-4 or anti-PD-L1 strengthened the treatment effect of the checkpoint blockade [155]. It has been shown that the simultaneous blockage of PI3k-γ and CSF-1R was able to reprogram TAMs into the M1-phenotype, activate the anti-tumor immune response, and further enhance the effect of anti-pancreatic cancer therapy [156]. Monophosphoryl lipid A (MPLA) is a TLR4 agonist. Recent studies reported that MPLA^+^IFNγ, both FDA-approved biological agents, reprogrammed TAMs to an M1-phenotype by stimulating type I IFN signaling and activation of cytotoxic T cells by IL-12 and TNFα secreted by macrophages. This led to a decreased tumor growth and inhibited metastasis in a mouse model of breast cancer and also enhanced the chemotherapy response in an ovarian cancer model [157].

### Inhibiting the recruitment or proliferation of TAMs and depletion of TAMs

Inhibiting monocyte recruitment into tumor tissue is a strategy to target TAMs. There are various ways for inhibiting a TAM recruitment and/or induce a TAM exhaustion, including inhibition of CSF-1R, blocking CCL2/CCR2, targeting CD40, and others. CSF-1, also named macrophage-CSF (M-CSF), stimulates colony formation [158, 159]. The administration of CSF-1R facilitated the progression and metastasis of tumors [160], the administration of CSF-1R inhibitor could defer tumor growth by changing the TAM polarization [161]. Therefore, CSF-1R could be explored as one of the molecules to target macrophages for cancer treatment [162]. CSF-1R inhibitors under clinical research or development include PLX3397, JNJ-40346527, ARRY-382, and BLZ945 [163–166]. Moreover, anti-CSF-1R antibodies including RG7155, IMC-CS4, and FPA008 are in a clinical evaluation stage [167]. In mouse glioma models, administration of a CSF-1R inhibitor (BLZ945) prolonged mouse survival and led to a shrinkage of established tumors [168]. Akkari et al. demonstrated in a mouse model that combining a CSF-1R inhibitor with radiotherapy to target glioma TAM populations, leads to a significant increase in the survival time [169]. Moreover, combining a CSF-1R inhibitor with a CXCR antagonist also resulted in a significant anti-tumor effect but more importantly, avoided granulocytes from infiltrating into tumors [170]. In addition, treatment with CSF-1R inhibitors and anti-PD-1 antibodies could induce melanoma to regress in a transplantation mouse model [171]. The results by Shi et al. indicate that a combination of a CSF-1R inhibitor (PLX3397), oncolytic viruses, and anti-PD-1 antibodies could enhance CD8^+^ T cell anti-tumor functions and prolong the survival of mice suffering from colon cancer [172]. Chai et al. showed that miR-26a expression inhibited the expression of CSF-1 and the infiltration of macrophages in HCC [173].

Targeting the CCL2/CCR2 axis is a perspective therapy for cancer. Blocking the CCL2/CCR2 axis by CCL2 knockdown or the administration of CCL2 inhibitors could significantly decrease tumor morbidity via preventing the recruitment and polarization of TAMs, thus enhancing the antitumor effect of CD8^+^ T cells in TME [174, 175]. Pienta et al. indicated that CCL2 blockade by administration of carlumab (CNTO88), inhibited the growth of prostate cancer cells and that the drug was well-tolerated by patients suffering from metastatic castration-resistant prostate cancer as shown in a clinical trial. Each treatment led to a transient decrease of serum CCL2 levels and some patients acquired a stable disease status [176]. Brana et al. found that a combination treatment of carlumab and standard chemotherapy was well-tolerated with only mild responses of side effects were observed [177]. Moreover, targeting CCR2 is also is an effective therapeutic strategy. The CCR2 inhibitor PF-04136309 could deplete the primary or premetastatic liver of TAMs by inhibiting CCR2^+^ monocytes mobilized from bone marrow to tumors, leading to a further enhanced anti-tumor immunity and the reduction of growth and metastasis of the tumor [178]. The study by Wu et al. illustrated that CCR2 inhibitors combined with the anti-PD-1 drug to treat cutaneous T-cell lymphoma reduced tumor growth [179].

CD40, a member of the TNF receptor superfamily, is expressed in antigen-presenting cells (such as dendritic cells) and facilitates the activation of anti-tumor T cells and the polarization of M1-phenotype cells [180]. The combination of CD40 agonists and anti-CSF-1R antibodies resulted in an increase in pro-inflammatory macrophages, dendritic cell maturation and differentiation. Moreover, this combination was able to eliminate the population that elicited the inhibitory immune response, thereby enhancing the antitumor response [181]. In addition, the results by Xiong et al. demonstrated that combining anti-CD40 treatment with anti-PD-L1 therapy, significantly increased the anti-tumor activity [144]. Anti-CD40 antibodies and recombinant CD40 ligands are currently being examined in clinical trials as a single agent or combination with chemotherapy/immunotherapy, for the latter e.g. with CP-870,893, RO7009789, APX005M, ADC-1013, SGN-40, and SEA-CD40 [182].

Trabectedin, through the TRAIL (also named TNFRSF10B) receptor (ET743, Yondelis®), targets TAMs by activating the caspase 8 cascade [183]. Trabectedin leads to a selective depletion of TAMs and is accompanied by a reduction in angiogenesis [183]. The drug was approved by the FDA in 2015 for the treatment of unresectable or metastatic liposarcomas or smooth muscle sarcomas [184]. Therefore, TAM reduction by stimulating TAM apoptosis through TRAIL receptors is a potential therapeutic strategy [185].

Decreased tumor burden and lung metastasis were observed after treatment with histone deacetylase inhibitor TMP195 that led to the recruiting of anti-tumor TAMs. Moreover, combination therapies of TMP195 together with chemotherapy or check-in point inhibitors could dramatically reduce the tumor burden compared to monotherapy with e.g. Carboplatin, Paclitaxel, and anti-PD-1 drugs [186].

### Others

A number of studies have demonstrated that nanoparticles can be employed as an effective therapeutic approach that may also be suitable as a new TAM-targeted strategy [187]. Hyaluronic acid-coated, mannan-conjugated MnO_2_ nanoparticles (Man-HA-MnO_2_ NPs) enabled repolarization of TAMs into an M1 phenotype (an anti-tumor phenotype) that significantly enhanced tumor oxygenation. At the same time, HIF-1 α and VEGF levels were downregulated, leading to a further relief of the tumor hypoxia and therefore the enhancement of the chemotherapy response. In addition, doxorubicin bound to Man-HA-MnO_2_ NPs was capable of synergistically checking the growth and proliferation of tumor cells [188]. The study by Penn et al. showed that G5-dendrimer nanoparticles loaded with methotrexate (as a ligand and toxin) was able to selectively target the folate receptor-2 (high expression in ovarian TAMs) on TAMs, leading to the depletion of TAMs in ovarian cancer tissues. Moreover, these results demonstrated that nanoparticles had a better therapeutic effect than cisplatin in cancer treatment [189]. Interestingly, investigators have also found that extracellular vesicles (e.g. exosomes) produced by M1-type macrophages can also reprogram TAMs to M1-like macrophages [187, 190]. It has been reported that M1-derived exosomes could strengthen the effect of delivering anti-tumor drugs and also improve the effectiveness of the treatment by releasing Th1-type cytokines [191]. Moreover, because of the pro-inflammatory character of M1-derived exosome content, they could be employed as a vaccine adjuvant [192]. However, due to the small number of extracellular vesicles produced by M1 macrophages and the complexity of isolation and purification, it is still a challenge at present [190, 193].

Zhang et al. designed a ultrasmall copper nanoparticles (Cu@CuOx) targeting CCR2, which could be loaded with the chemotherapy drug gemcitabine, and that could be delivered to PDAC tissues. Cu@CuOx was shown to significantly inhibit tumor progression and also to prolong survival in a syngeneic xenograft mouse model [194].

The immunosuppressive phenotype of TAMs is mainly determined by the unsaturated fatty acid metabolism which converts bone-marrow-derived macrophages to the M2 phenotype. Lipid droplets enriched in this process could therefore serve as a target for chemical inhibitors that block TAM polarization and tumor growth [195].

The above results indicate that targeted TAM therapies are potential strategies for cancer treatment. However, the development of an appropriate treatment strategy depends on the tumor type and the role of targeted TAMs as well as the application of appropriate therapeutic agents.

## Conclusion and perspective

It is commonly agreed upon that macrophages are crucial for the initiation and progression of tumors. These macrophages are divided into two types: M1-like and M2-like phenotypes. M1 has been characterized as being pro-inflammatory and able to phagocytize tumor cells, while M2 has been characterized as being anti-inflammatory. TAMs are considered as belonging to the M2-phenotype that can facilitate tumor initiation, angiogenesis, invasion and metastasis. An increasing number of studies have revealed the influence of the TME on tumorigenesis and development. TAMs as a major component of the TME, are a complex heterogeneous cell population that contributes to the malignant character of solid tumors. The heterogeneity and specific characteristics of TAMs in tumors lay a foundation for the development of personalized TMA-based treatment methods. We summarized the current major therapeutic approaches for targeting TAMs, for example, targeting angiogenesis, inhibiting the recruitment of TAMs, and reprogramming TAMs. It is worth mentioning that nanoparticle development is becoming increasingly popular and that these nanosystems can be employed as drug carriers. In this paper, we briefly mentioned the carrier role of nanoparticles or extracellular vesicles in TAMs and presented the current limitations. Given the complex role of TAMs in tumorigenesis, we need more in-depth studies on their functions and the regulatory mechanisms to discover effective anti-tumor targets. Targeting TAMs may result in reversing the TME with regard to tumor promotion and immune inhibition, a promising novel therapeutic modality for future precision tumor treatments. Besides, experimental, preclinical, and clinical studies on TAMs are ongoing and we believe that targeting TAMs will be a valuable therapeutic strategy in the future. There are still many unanswered questions about the key molecules or signals functionally reprogramming TAMs. Further understanding of the regulation of TAMs is required to answer these questions. In conclusion, TAMs are functionally diverse and play complex roles in the TME. Targeted mono-therapy of TAMs or in combination with conventional therapeutic approaches may shed more light on future options of cancer treatment.

## Data Availability

Not applicable.

## Abbreviations

TAM: Tumor-associated macrophage
TME: Tumor microenvironment
CCL2: C–C chemokine ligand 2
CCL5: C–C chemokine ligand 5
CSF-1: Colony-stimulating factor-1
GM-CSF: Granulocyte–macrophage colony-stimulating factor
NSCLC: Non-small cell lung cancer
EMP: Erythroid-myeloid progenitors
MDP: Monocyte-producing macrophage/dendritic progenitors
VEGFA: Vascular endothelial growth factor A
CCL18: C–C chemokine ligand 18
CCL20: C–C chemokine ligand 20
HCC: Hepatocellular carcinoma
CXCL12: C-X-C motif chemokine ligand 12
IFN-γ: Interferon-γ
TNF-α: Tumor necrosis factor-α
LPS: Lipopolysaccharide
Th: Helper T cell
iNOS: Nitric oxide synthase
ROS: Reactive oxygen species
IL-12: Interleukin-12
IL-4: Interleukin-4
IL-10: Interleukin-10
IL-13: Interleukin-13
CD68: Cell differentiation 68
CD163: Cell differentiation 163
CD206: Cell differentiation 206
NK cells: Natural killer cells
NF- κB: Nuclear factor- κB
STAT3: Signal transducer and activator of transcription 3
HIF1 α: Hypoxia inducible factor 1 α
EGF: Epidermal growth factor
TGF-β: Transforming growth factor
COX-2: Cyclooxygenase-2
PGF: Placenta growth factor
FGF: Fibroblast growth factor
PDGF: Platelet-derived growth factor
Ang: Angiotensin
IDO: Indoleamine 2,3-dioxygenase
MMP-9: Metalloproteinase-9
ECM: Extracellular matrix
EMT: Epithelial-mesenchymal transformation
CRC: Colorectal cancer
PRL-3: Phosphatase of regenerating liver-3
ERO 1-α: Endoplasmic reticulum oxidoreductase 1-α
CRYAB: αB-Crystallin
MDSCs: Bone marrow-derived suppressor cells
Treg cell: Regulatory T cell
PD-1: Programmed cell death 1
PD-L1: Programmed cell death ligand 1
ADCP: Antibody-dependent cellular phagocytosis
ADCC: Antibody-dependent cellular cytotoxicity
TRAIL: TNF-related apoptosis-inducing ligand
CSC: Cancer stem cell
HA: Hyaluronic acid
MFG-E8: Milk-fat globule-epidermal growth factor-VIII
SIRP α: Signal-regulatory protein α
CAR-T cells: Chimeric antigen receptor T cells
TLRs: Toll-like receptors
MPLA: Monophosphoryl lipid A
PAMPs: Pathogen-associated molecular patterns
PDAC: Pancreatic ductal adenocarcinoma
Man-HA-MnO2 NPs: Mannanconjugated MnO2 nanoparticles
