# Correction to: Roles of tumor-associated macrophages in tumor progression: implications on therapeutic strategies

**DOI:** 10.1186/s40164-022-00258-1

**Published:** 2022-02-03

**Authors:** Shuangli Zhu, Ming Yi, Yuze Wu, Bing Dong, Kongming Wu

**Affiliations:** 1 Department of Oncology, Tongji Hospital of Tongji Medical College, Huazhong University of Science and Technology, Wuhan, 430030 China; 2 Department of Molecular Pathology, The Affiliated Cancer Hospital of Zhengzhou University & Henan Cancer Hospital, Zhengzhou, 450008 China

## Correction to: Experimental Hematology & Oncology (2021) 10:60 https://doi.org/10.1186/s40164-021-00252-z

Following publication of the original article [1], the author reported error in first author name. The first author name was misspelling in the submitted version and published as Shangli Zhu. The correct version is “Shuangli Zhu”.

The original article has been corrected.
